# Critical Challenges in Cancer Immunotherapy and Cardiac Health

**DOI:** 10.2174/011573403X353300250407090657

**Published:** 2025-04-23

**Authors:** Aftab Alam, Sushma Devi, Sana Hashmi

**Affiliations:** 1 Department of Pharmacognosy, College of Pharmacy, Prince Sattam Bin Abdulaziz University, Al Kharj 11942, Saudi Arabia;; 2 Chitkara College of Pharmacy, Chitkara University, Rajpura, 140401, Punjab, India;; 3 Department of Pharmaceutical Sciences, Unaizah College of Pharmacy, Qassim University, Unaizah 51911, Saudi Arabia

**Keywords:** Cancer immunotherapy, immune checkpoint inhibitors, immune-related adverse events, cardiovascular risk, artificial intelligence, cardiac monitoring

## Abstract

Cancer immunotherapy is based on immune checkpoint inhibitors (ICIs) and has brought a revolution in oncology with promising treatment possibilities for diverse cancers. Yet, immune-related adverse events (irAEs) frequently limit the clinical efficacy of ICIs, with cardiotoxicity representing a significant salient consequence. These ICI side-effects underscore the need for well-established models for the assessment of cardiac risk. This review proposes a risk evaluation strategy that uses biomarkers, non-invasive imaging, and individual patient data. The goal is to elucidate the mechanism through which immune-related adverse events affecting the heart might arise, and the need for predictive tools to better tailor treatment regimens to increase both safety and efficacy. Biomarkers play a vital role in the detection and prevention of heart-related side effects,  which means adequate intervention while preserving therapeutic outcomes. Moreover, the study discusses the acknowledgement of novel treatment regimens and the ability of integration of artificial intelligence (AI) and machine learning (ML) to improve the assessment of risk. AI/ML tools are experts at synthesizing heterogeneous datasets to reveal patterns and risk factors, providing clinicians with powerful capabilities to enhance safety and efficacy. This paper aims to develop sound risk assessment models to enhance both the safety and efficacy of cancer immunotherapies by exploring various strategies and interactions in immunotherapy.

## 1 INTRODUCTION

Cancer continues to pose a health challenge as conventional treatments frequently prove inadequate in terms of effectiveness and patient well-being. In 2020, about 19 million people were diagnosed with cancer, and about 10 million cancer deaths were estimated [1]. According to such projections, by the year 2040, there will be a sharp increase in the incidence of cancer, with 20.7 million new cases and 12.7 million deaths among older individuals [2]. This is evidence of the growing need for new therapeutic strategies to combat this urgent challenge.

Immunotherapy has pioneered a new era of cancer treatment by harnessing the immune system to identify and destroy cancer cells. Different from traditional therapies like chemotherapy and radiation that directly attack cancer cells, immunotherapy is making the most of the immune system. This is not chemotherapy or radiation; it is empowering the immune system to fight off cancer cells. Immunotherapy has demonstrated activity across many different cancers, including melanoma, lung cancer, and hematologic malignancies [3]. One of the most revolutionary immunotherapy strategies is immune checkpoint inhibitors (ICIs), which inhibit regulatory molecules such as cytotoxic T-lymphocyte antigen 4 (CTLA-4) and programmed death protein 1 (PD-1) or its ligand (PD-L1). These kinds of inhibitors boost the immune system’s ability to attack tumours, achieving durable responses in a variety of malignancies, including melanoma, lung cancer, and hematologic cancers [4].

The success of ICIs has ushered in a paradigm shift in cancer therapy towards immune-based precision medicine. Table **1** summarizes the comparison of immune checkpoint inhibitors *versus* conventional therapies (including chemotherapy and radiation) in mechanistic, efficacy, and safety aspects [5-16].

Nevertheless, the incredible therapeutic promise of ICIs is balanced by immune-related adverse events (irAEs), seen in 70–90% of patients [17]. Such complications result from systemic immune activation and can involve multiple organs, including the cardiovascular system [18]. Cardiovascular irAEs, although rare themselves, pose special concerns with high mortality, at rates from 20% to 45%, including pericarditis, vasculitis, and myocarditis [19]. These conditions are heterogeneous and can be clinically characterized as either asymptomatic biomarker elevations or severe acute presentations that necessitate prompt intervention. Although the exact pathophysiology of these cardiac irAEs is not clearly defined, do appear to involve direct immune-mediated inflammation, cytokine release, and potential autoantibody generation [20, 21].

The rising awareness of cardiac irAEs reflects the need for an overall approach to minimizing the disadvantages without losing the advantage of ICIs. Early detection of myocardial damage and inflammation may be aided by wet biomarkers such as cardiac troponins and B-type natriuretic peptides (BNP), yet their clinical utility remains to be proven [22, 23]. Additional non-invasive techniques including computed tomography (CT), nuclear medicine, echocardiography, and magnetic resonance imaging (MRI) provide vital data about structural and functional alterations of the heart, crucial for characterizing disease evolution over time [24]. The multicomplex and multifactorial character of cardiac irAEs suggests that the use of biomarkers and imaging alone may not be enough for the proper management of relevant patients.

In response to these obstacles, artificial intelligence (AI) and machine learning (ML) are quickly materializing as essential technologies to improve early detection, risk stratification, and personalized treatment strategies. AI/ML algorithms demonstrated the capability to reveal convoluted configurations and prognosticate deleterious consequences of employing a diverse random of data with different source types including biomarkers, imaging, and clinical parameters, and outperform conventional methodologies, where extensive datasets may not have yet become accessible [25-27]. Such integration will improve treatment protocols, ensure optimization of therapeutic decision-making, and equality of efficacy and safety in cancer immunotherapy.

This review discusses the major concerns related to ICIs in the cardiac field, highlighting the need for novel diagnostic tools and personalized treatment strategies. Biomarkers, non-invasive imaging techniques, and computational tools like AI and ML may augment our ability to detect relatively modest elevations in risk of these important adverse events (cardiac irAEs) earlier and more accurately. The use of these technologies in clinical protocols could help develop treatment regimens, improve patient safety, and allow for the continued efficacy of cancer immunotherapy.

## 2 IMMUNE CHECKPOINT INHIBITORS AND CARDIOTOXICITY

Immune checkpoints are essential in preserving immune homeostasis, including CTLA-411, PD-112, and PD-1 ligand PD-L1. CTLA-4 is involved in the regulation of T cell activation within lymph nodes, while PD-1 pathways modulate immune responses later in the peripheral tissues. When ICIs block these pathways it keeps the T-cells activated and proliferative, making it possible for the body to effectively combat and eradicate tumours *via* the immune system [28]. ICIs have completely transformed the treatment of cancer diseases by boosting the immune system's response to cancer cell attacks. But they can also lead to irAEs that may impact the immune system itself. These adverse reactions have varying degrees of severity from minor to rare and serious (including pericardial diseases and vasculitis) [29]. The transition from early initial ICI therapy to the emergence of cardiac toxicities is displayed in Fig. (**1a**) [30].

**Fig. (1):**
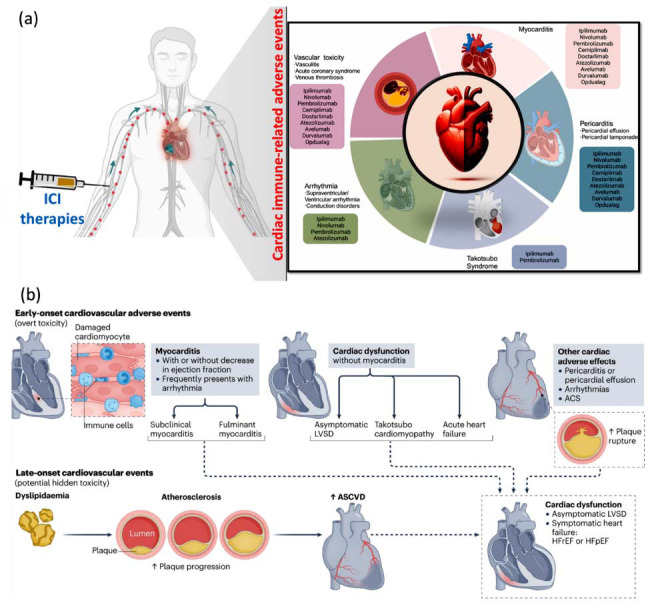
(**a**) The spectrum extends from initial immune checkpoint inhibitor therapy to the development of cardiac toxicities [30]; (**b**) Clinical presentations of immune checkpoint inhibitor-induced cardiotoxicity, whether early-onset or late-onset [31]. **Abbreviations:** ICI therapies: Immune checkpoint inhibitor therapies; LVSD: Left ventricular systolic dysfunction; ASCVD: Atherosclerotic cardiovascular disease; HFpEF: Heart failure with preserved ejection fraction; ACS: Acute coronary syndrome.

With ICI treatment comes the risk of irAEs, myocarditis being the most common initial cardiac manifestation studied. It has a mortality rate of around 50%. ICI-induced myocarditis clinically can have a variable presentation ranging from mild to severely symptomatic with arrhythmias and/or cardiac dysfunction. These could vary from left ventricular systolic dysfunction to acute cardiac failure. The development of cardiovascular events is also associated with the impairment of arterial atherosclerosis, therefore increasing the risk of heart disease. Late-onset cardiac dysfunction can either be asymptomatic and present as a decrease in ejection fraction or symptomatic heart failure (Fig. **1b**) [31].

However, this phenomenon can be well illustrated by a case study in the literature. In a cohort of more than 100,000 individuals with cancer, a portion of those received treatment consisting of ICIs. Quinlan *et al.*, 15 in a retrospective review of 80 patients, found complications in 14.6% of patients after initiation of therapy. Heart failure and/or cardiomyopathy occurred in 5.6% of patients treated with a single ICI (*e.g.*, PD-1 or PD-L1) and in 6.1% of those treated with combination ICI therapy (*e.g.*, PD-1 and CTLA-4). Cardiac complications usually occurred around 63 days after the start of the therapy. One case of myocarditis occurred 28 days after nivolumab was administered. Moreover, patients diagnosed with newly developed conditions during ICI treatment had significantly lower survival rates than those who did not develop immunotherapy-associated complications, with a mortality rate of 66.1% *vs* 41.4% [32].

The basis of cardiac irAEs is the uncontrolled response of T cells, usually protective, against heart muscle tissue. This aberrant activation is predominantly induced by ICIs that inhibit signals inhibiting T cell activity, causing indiscriminate immune activity. Activated T cells invade the myocardium and secrete proinflammatory cytokines such as pro-inflammatory cytokines, including interferon-gamma (IFN-γ) and tumour necrosis factor-alpha (TNF-α). These receptors act to spur inflammation, but at high levels, they contribute to tissue damage and recruit more immune cells, creating exaggerated damage [33, 34]. At the molecular level, key immune components are engaged in the pathogenesis, resulting in inflammation with cardiac injury by different mechanisms.

### 2.1 T-cell Activation and Infiltration

The precise mechanisms of immune-related cardiac toxicity remain not fully elucidated. The latter, however, are believed to be mediated by immune checkpoints, which are physiological mechanisms that regulate immune homeostasis. Immune checkpoints are inhibitory co-receptors on T cells that modulate immune responses while limiting autoimmunity [35]. T cells fall into two broad categories: CD4+ helper T cells and CD8+ cytotoxic T cells. CD4+ helper T-cells are known to aid the immune response through antibody class-switching in B-cells, which can induce loss of immune tolerance. They also stimulate CD8+ cytotoxic T-cells through interactions with dendritic cells and work to enhance the phagocytic activities of macrophages and neutrophils. In contrast, CD8+ cytotoxic T cells act primarily to destroy target cells, including infected or neoplastic cells. These different functions of T-cells play a role in the global immune response and may indulge immune-related cardiac toxicity [29, 36].

The heart has its own immune system, where T cells are usually present in reduced numbers when not activated [37]. Heart-resident macrophages and dendritic cells present antigens through major histocompatibility complex (MHC) class I or II molecules to T cell receptors (TCRs), and the lectures TCRs require co-stimulation for activation. Balancing activation status for T cell activity is mediated by stimulatory and inhibitory signals. In addition, immune checkpoints, including CTLA-4, PD-1, PD-L1, and Lymphocyte Activation Gene 3 (LAG-3), critically govern immune tolerance and the prevention of autoimmune attacks on cardiac tissue,  mediating T cell responses in health and disease [29]. Both normal and pathologic heart sites can produce immune checkpoints such as CTLA-4, PD-1/PD-L1, and LAG-3 for the modulation of T cells. These checkpoints serve as critical regulators of immune tolerance and the prevention of autoimmune responses within cardiac tissues.

Systemic balance is achieved through checkpoints regulating T-cell activation. PD-1/PD-L1 signalling is critical for preventing T cells and maintaining balance and preventing cardiac tissue autoimmune responses [38]. A-Cardiac T-lymphocyte infiltration and accumulation correlates with multiple immune-mediated cardiac diseases. Inflammation of the myocardium prompts an auto-response from the body to upregulate the levels of some local immune markers, which serve to reduce inflammation in the heart. However, cancer treatments that aim to suppress these immune markers may interfere with this protective response, thereby raising the risk of serious heart damage [30]. In myocarditis, T-cells mistakenly attack heart tissue, leading to inflammation that interferes with heart function [39].  Chronic T-cell activation in the heart is responsible for cardiac remodelling and dysfunction in dilated and ischemic cardiomyopathies, thus contributing to disease severity [40]. This inflammation can damage heart muscle cells, draining the heart’s strength and endangering heart failure [41].

**Fig. (2):**
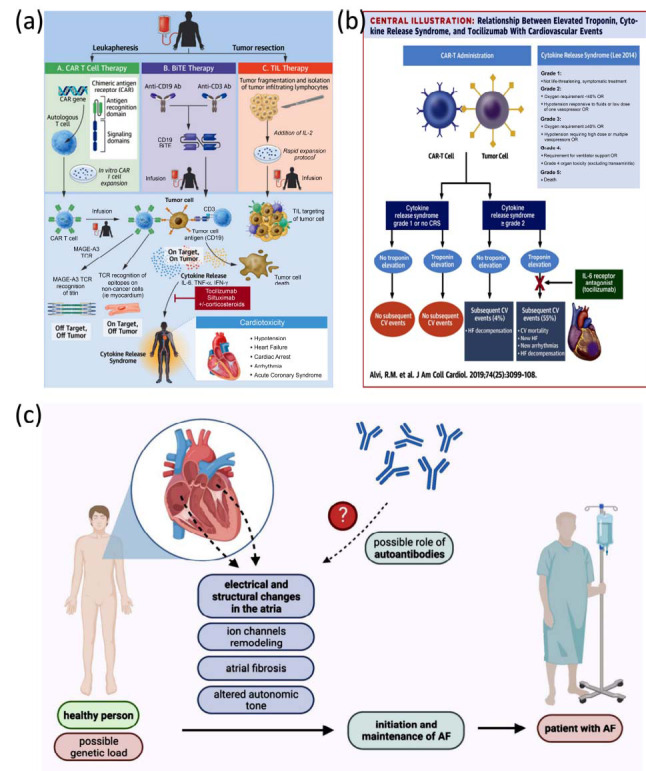
(**a**) Mechanisms of T-cell therapies and manifestations of cardiotoxicity [42]; (**b**) Relationship between elevated troponin, cytokine release syndrome, and tocilizumab with cardiovascular events [43]; (**c**) Pathological mechanisms of AF initiation and maintenance with the possible role of autoantibodies [44]. **Abbreviations:** CAR T Cell Therapy: Chimeric Antigen Receptor T Cell Therapy; BITE Therapy: Bispecific T Cell Engager Therapy; TIL Therapy: Tumor-Infiltrating Lymphocyte Therapy; Ab: Antibody; MAGE-A3 TCR: Melanoma-Associated Antigen A3 T Cell Receptor; IL-2: Interleukin-2; CRS: Cytokine Release Syndrome; CV events: Cardiovascular events; IL-6: Interleukin-6; AF: Atrial Fibrillation.

While T cells help the heart repair itself, they can also contribute to its damage in heart injury models such as myocarditis and cardiomyopathy. By utilizing checkpoints to modulate T cell activity, the heart is free from damage (Fig. **2**) [42-44]. Cancer immunotherapies that target these checkpoints may disturb this balance, with the potential for cardiac damage as illustrated in Fig. (**2a**).

### 2.2 Cytokine Release

Cytokine release syndrome (CRS) is a common inflammatory phenomenon associated with chimeric antigen receptor (CAR) T-cell therapy, and occurs in approximately 42% to 100% of patients, including severe CRS in 0% to 46% of patients following CAR T-cell infusion [45, 46]. The proliferation of T cells during the process of CAR T-cell therapy triggers the secretion of a variety of cytokines intended to target neoplastic cells, but those cytokines can also affect cardiac tissues,  which may lead to cardiotoxicity [47, 48]. Many essential pro-inflammatory cytokines, including TNF-α and IFN-γ, are responsible for promoting inflammatory responses and may contribute to cardiac inflammation [49]. Moreover, prominent cytokines like interleukin-1 (IL-1), IL-6, and IL-17 drive inflammatory responses and are implicated in the pathogenesis of various autoimmune and inflammatory diseases [50]. The cardiac-related responses underscore the potential risks of immunotherapy even with CAR T-cell therapies. Hence, CRS is another significant adverse event of CART cell therapy, and its consequences and accompanying cardiotoxicity demand close surveillance and management [51].

Like high-dose IL-2 therapy induces increased vascular permeability in a dose-dependent manner resulting in vascular leak syndrome (VLS). This syndrome mainly affects the lungs causing symptoms, like edema and similar symptoms. The pathophysiological processes include the release of cytokines, activation of endothelial cells, and infiltration of leukocytes, which lead to disruption of the alveolar-epithelial barrier and vascular leakage [52, 53]. Although IL-2 enhances T-cell function, it is associated with vascular leakage and cardiovascular toxicity. Increased IL-1α expression is associated with fever and systemic inflammatory responses, important hallmarks of CRS [54]. Higher IL-17 was linked to increased inflammation and tissue damage, which implicates it in CRS-related cardiotoxicity [55].

Cytokine-induced cardiac dysfunction has been demonstrated in animal models. For instance, IL-2 administration in mice results in myocardial fibrosis and heart failure, emphasizing its cardiovascular off-target activities [56]. Biomarkers such as troponin are frequently monitored in clinical settings to assess cardiac health during CRS induced by ICI therapy. In these patients raised troponin levels show possible myocardial injury, which could be due to direct inflammation or as a result of a collateral condition like hypotension or arrhythmias. Importantly, previous studies have demonstrated cases of elevated troponin levels in individuals receiving nivolumab treatment with no apparent etiology for these elevations and higher rates of subclinical myocarditis [57].

Sarocchi *et al*. reported unexplained elevated troponin levels in 10% of patients treated with nivolumab, possibly indicating an increased incidence of subclinical myocarditis [58]. This highlights the importance of cardiac monitoring in immunotherapy. Tocilizumab, an IL-6 receptor antagonist is effective for managing the CRS, a condition associated with cardiovascular complications. Ferreros *et al*. have shown that the cardiovascular outcomes and mortality were significantly lower with early tocilizumab treatment in patients with elevated troponin and CRS [59]. The relationship between increased troponin, CRS, and cardiovascular events associated with tocilizumab is described in Fig. (**2b**) [43]. These findings emphasize the need for careful cardiac monitoring and early intervention in patients receiving IL-2 or other immunotherapies to help manage the potential cardiovascular risks adequately.

### 2.3 Autoantibody-mediated Cardiotoxicity

The emergence of autoantibody-mediated cardiotoxicity during ICI therapy has raised concerns in the field of cancer immunotherapy [60]. With this immune activation, which is clinically associated with tumor cell killing, one might also get autoantibody production, which is also clinically associated with irAEs. During ICI therapy, autoantibodies (immune proteins that erroneously mount an attack on the body’s tissues) occur as a result of improper immune checkpoint. These enhanced immune responses may lead to collateral attack on cardiac tissues, contributing to cardiotoxicity [61]. Mechanistically, tumour antigens that share structural or sequence similarity with cardiac proteins may drive cross-reactive immune responses, as seen for cardiac myosin and mitochondrial antigens. This tissue mimicry activates T-cells and leads to the production of autoantibodies that go after the cardiac tissues. The result, in addition to tumour attack, is heart damage, resulting in myocarditis and pericarditis. The occurrence of cardiac irAEs in the context of these events points to the possible involvement of shared epitopes between tumour and cardiac antigens in the pathogenesis of the cardiac irAEs [20, 27].

The association of selected autoantibodies with severe cardiac irAEs is demonstrated in clinical studies. To illustrate, anti-striated muscle and anti-mitochondrial antibodies have been associated with myocarditis and pericarditis, diseases that confer significant morbidity and mortality. Rubio-Infante *et al.* have provided insight into the interplay between autoimmunity and cardiotoxicity, emphasizing the role of autoantibodies in cancer immunotherapy [62]. In addition, patients treated with anti-PD-1 and anti-PD-L1 therapies are at a higher risk of developing myocarditis, which has been especially noted in patients who present with myositis, further linking the role of autoantibodies such as anti-striated muscle and anti-mitochondrial antibodies [63].

The characteristic mechanisms of action of ICIs manifest in their highly different toxicity profiles. Compared with ipilimumab, a CTLA-4 inhibitor, nivolumab, a PD-1 inhibitor, is associated with a lower incidence of overall irAE. Nivolumab, though, has its own associated autoantibody-mediated toxicities, such as myocarditis, myositis, and myasthenia gravis [49]. For instance, Suzuki *et al*., myasthenia gravis (MG) was identified in 0.12% of patients treated with nivolumab, frequently in the setting of overwhelming myositis and myocarditis [64]. Ipilimumab, in contrast, carries a higher overall rate of irAEs, including colitis and endocrine dysfunction, but a lower association with cardiac irAEs. Combination regimens with nivolumab and ipilimumab markedly increase the risk of high-grade irAEs with myocarditis [65, 66]. It serves as a foundation for a better understanding of autoantibody-mediated cardiotoxicity and improvement in clinical outcomes. Autoantibody screening and tracking emergence during therapy may enable the identification of patients at risk for cardiac irAEs. Investigating the similarities of tumor-cardiac antigens might promote damage mitigation without compromising the antitumor efficacy of ICIs as shown in Fig. (**2c**). As such, cardiac irAEs management comprises symptom monitoring, cytokine and autoantibody evaluation, and immunosuppressive therapy in severe situations. Although difficult, new research and clinical strategies can provide hope.

In summary, elucidation of the molecular and immunological mechanisms underlying cardiac irAEs is an important area of investigation for both research and clinical practice. More studies are needed to help explain the complete spectrum of immune-related events in those receiving ICIs, and to create therapies we can use to prevent these potentially deadly, immune-related complications. These approaches seek to restore the controlled antitumor potential of the immune system while protecting harmful autoimmune reactions, ultimately improving the safety and efficacy of cancer immunotherapy.

## 3 CHALLENGES IN EARLY DETECTION OF CARDIAC IRAES

During cancer treatment with ICIs, healthcare professionals face challenges in detecting irAEs. ICIs targeting CTLA-4, PD-1, and PD-L1 improve tumour immunity but also induce a broad spectrum of irAEs, including rare but life-threatening cardiovascular complications such as myocarditis [67, 68]. ICIs can alter immune homeostasis leading to inflammatory responses that may involve the heart. Myocarditis, arrhythmias, and heart failure are examples of cardiac irAEs that are difficult to diagnose due to nonspecific symptoms. Earlier diagnosis and prompt management are crucial in ICI-associated myocarditis with a high mortality rate (up to 50%) [69]. The management of ICI-related myocarditis usually requires the prompt discontinuation of ICI therapy and the institution of high-dose corticosteroids to suppress the inflammatory response. Patients with severe cases may also need additional immunosuppressive therapies. Multidisciplinary treatment paradigms with the engagement of oncologists, cardiologists, and immunologists are necessary to improve patient outcomes. Timely recognition is critical as treated occult infections have a higher risk of mortality [70].

Diagnosis of cardiac irAEs usually requires peripheral fluid analysis (troponins) and ECGs; echocardiography, cardiac MRI, and occasionally a biopsy may be required [71]. These need to be closely monitored by a cardiologist to help with decisions on whether or not to stop treatment. For cardiac irAEs, glucocorticoids represent the mainstay of therapy and are often escalated to other immunosuppressive medications in more advanced cases [72]. Common symptoms indicative of cardiac irAEs, including fatigue, chest discomfort, and shortness of breath, are non-specific and can result from multiple non-cardiac medical conditions, which creates diagnostic challenges [73].

The overlap of symptoms and timing makes it difficult to distinguish cardiac irAEs from other conditions. To diagnose it on time, we need a holistic approach that combines clinical acumen, imaging, and biomarker assessment for efficient diagnosis and treatment. The diagnosis of cardiac irAEs relies heavily on advanced imaging techniques. ECG is useful for the detection of arrhythmias and for myocardial injury, whereas echocardiography helps assess the integrity of the structure and function of the heart [70, 74]. Both cardiac MRI, which is best known for its ability to assess myocarditis, and other abnormalities, and cardiac CT, which can evaluate coronary vessels involved during certain toxicities, are essential in diagnosing immune-related cardiac events [75]. While these are indispensable imaging modalities, they might not always facilitate the detection of subtle pathological alterations and underscore the complementary role of biomarker evaluations as depicted in Fig. (**3a**) [76].

Novel emerging biomarkers may play a role in the early detection of immune checkpoint inhibitor-related myocarditis. Pretreatment molecular profiling of DNA, mRNA, proteins, and cells from blood may extract meaningful information for predicting cardiac sequelae of immunotherapy [77]. Advancements in biomarker research could lead to innovative screening approaches, allowing timely detection and treatment to enhance patient prognosis and survival rates [78]. Imaging studies alone cannot detect subtle pathological changes or provide real-time monitoring of the disease. The use of biomarkers would present an opportunity to improve diagnostic specificity and promote early detection of cardiac irAEs.

Newly developed biomarkers have shown promise in addressing these diagnostic obstacles. Troponin, B-type natriuretic peptide (BNP) [74], and C-reactive protein (CRP) [79] are a few categorically defined biomarkers that are useful in identifying myocardial injury and inflammation. Incorporating biomarker evaluations into clinical settings allows for earlier identification of patients and helps in initiating early intervention, with the potential to improve clinical outcomes. Troponin is a well-used marker for myocardial damage, while BNP and CRP assess cardiac stress and systemic inflammation, respectively [80]. Biomarker panels that are also able to detect different aspects of cardiac dysfunction improve diagnostic accuracy and guide personalized treatment plans. These panels have the potential to modernize differential diagnosis and risk stratification for cardiac irAEs, leading to a personalized medicine approach in cardiology. The potential role of imaging and molecular-level biomarkers in the timely detection and monitoring of cardiac irAEs are well described in Fig. (**3b**) and opens the door for their use in the future [81].

Hence, biomarkers are revolutionizing cardiac by allowing for early detection, individualized treatment, and improved outcomes, especially for cardiac irAEs. A combination of the diagnostic methods, such as biomarker panels, clinical assessment imaging, facilitate timely and appropriate management of irAEs, overcoming challenges related to nonspecific clinical features and severe complications. This emphasizes the importance of precision medicine in enhancing cardiology and optimizing therapy in the new age of cancer immunotherapy.

## 4 ROLE OF BIOMARKERS IN CARDIAC IRAES MANAGEMENT

Biomarkers are also essential for the identification, risk assessment, and management of cardiac irAEs associated with ICI therapy. Based on the above three subdivisions, the biomarkers can also be divided into three major types: first, inflammatory biomarkers, second, cardiac injury biomarkers, and last, emerging biomarkers, which provide additional pathophysiological relevance and potential implications on the ongoing utility of cardioprotective medications in patients developing cardiac irAEs.

### 4.1 Inflammatory Biomarkers

Biomarkers of inflammation (*i.e*., C-reactive protein [CRP], cytokines) play a critical role in detecting systemic inflammation related to irAEs. While previous studies demonstrated a strong correlation between high levels of high-sensitivity CRP (hs-CRP) with increases in cardiovascular and inflammatory processes in patients receiving ICI therapy, the actual mechanisms behind this phenomenon remain poorly understood [82]. For example, work by Onodera and colleagues carried out a research study to assess whether CRP levels prior, to receiving ICI treatments could act as indicators for both the development of irAEs and the overall survival (OS) of patients with non-small cell lung cancer (NSCLC). They retrospectively analyzed data from NSCLC patients who received ICIs at Iwate Medical University Hospital categorizing them into high CRP groups based on a cut-off value of 10 mg/L. The analysis showed an incidence of irAEs, severe cases in the high CRP group compared to the low CRP group with pneumonitis being the most common severe irAE. Multivariate analysis indicated that elevated CRP levels were associated with an increased risk of developing irAEs. Additionally, patients with CRP levels had progression-free survival (PFS) and OS compared to those with low CRP levels. These findings suggest that elevated pre-treatment CRP levels may indicate susceptibility to irAEs and a favorable prognosis in NSCLC patients receiving ICI therapy highlighting the use of CRP, as a predictive marker [83]. Along with CRP, cytokines are presented in the development of myocarditis, where they may provide even more information regarding the degree of immune dysregulation severity [84, 85]. Such biomarkers are informative not only of active inflammation but also play prognostic roles, aiding clinical outcome prediction.

### 4.2 Cardiac Injury Biomarkers

Biomarkers of cardiac injury, such as cardiac troponins and natriuretic peptides, are useful for diagnosing myocardial injury and dysfunction in patients with cardiac irAEs. Cardiac troponin I (cTnI) and cardiac troponin T (cTnT) have become preferred biomarkers for detecting myocardial injury because of their cardiac-specific isoforms, offering high clinical sensitivity and specificity [86-90]. The elevation of cardiac troponin levels signifies myocardial necrosis, a characteristic feature of myocarditis. Diagnosing myocarditis induced by ICIs proves challenging due to the variability of symptoms and the potential rapid progression of the condition. Identifying elevated cardiac troponin levels plays a pivotal role in diagnosing this condition, prompting further assessments and the initiation of treatment. Westwood *et al*. stress the importance of early detection of these elevations, as it facilitates the prompt initiation of treatment, thereby significantly improving patient outcomes [91]. Exploring more deeply the pathophysiology of ICI-triggered myocarditis and investigating the kinetics of troponin release could improve the diagnostic and prognostic potential of these crucial cardiac biomarkers. For instance, troponins are well-known markers found in the blood and are commonly used to detect heart muscle damage. They tend to be higher in patients, with heart inflammation or other heart-related issues [87]. Cardiac troponins are a type of protein that plays a role in facilitating the interaction, between actin and myosin in contracting cardiac muscles with calcium acting as the mediator [88]. Usually, the blood doesn't show these proteins. However, when there’s a heart injury the heart cells release them into the bloodstream. This makes them great markers, for heart damage [89]. In a similar vein, natriuretic peptides like BNP [92] and NT-proBNP (N-terminal pro-B-type natriuretic peptide) are utilized to indicate myocardial strain and heart failure [74]. For instance, the predictive capability of BNP for stroke risk in patients with heart failure and preserved ejection fraction (HFpEF) was explored by Liu *et al*. Data from 799 HFpEF patients in the TOPCAT trial were analyzed, revealing that elevated baseline BNP levels correlated with a heightened stroke risk. The inclusion of BNP in current stroke risk assessment scores notably enhanced their predictive accuracy for stroke [93]. Cytokines drive immune responses in ICI-related cardiac complications. IL-8, IL-6, granulocyte-macrophage colony-stimulating factor (GM-CSF), and interferon-γ promote inflammation, immune cell activation, and tissue damage, leading to myocarditis and cytokine release syndrome [94].

Biomarkers offer a strategic tool to relay the management of cardiac irAEs in patients receiving ICIs. These key markers including CRP; IL-6; cardiac troponins and BNP allow for the timely detection, risk stratification, and timely intervention allowing the opportunity to prevent progression to severe disease, such as myocarditis. New strategies, such as multiplex biomarker panels, advanced imaging, and integration with AI-based platforms, are improving diagnostic accuracy and risk stratification.

A biomarker-guided strategy is not only a new way to approach a cardiac irAE but also ensures potentially life-saving ICI therapies are delivered without compromising cardiac health. As research into novel biomarkers and advanced diagnostic technologies continues, the future of heart failure management will likely see even more refined approaches to patient care and the further evolution of personalized medicine in this complex and evolving space [84].

## CONCLUSION

Anticancer immunotherapy has revolutionized the field of oncology, providing effective approaches beyond standard therapies, including chemotherapy and radiation. ICIs have been especially transformative, using the body’s immune system to find and kill cancerous cells. While the introduction of irAEs, particularly cardiotoxicity, has exposed some important challenges.

Such issues would need to be addressed through a process of forward-looking integration of biomarkers along with AI and ML which is highly essential for enhancing the safety, efficacy,  and personalization of the immunotherapy process. Biomarkers of irAEs in cancer immunotherapy- a window for prediction and understanding. Although traditional markers like cardiac troponins spotlight cardiac tissue injury, novel multiparametric panels encompassing genomic, proteomic, and metabolomic changes can provide more extensive risk assessment. Genomics exposes the genetic basis of predisposition, while (mass-spectrometry-based) proteomics and metabolomics expose the immune dysregulation that is amenable to early detection and accurate stratification.

A transformative development on the horizon is the real-time monitoring of biomarkers. Wearable biosensors and lab-on-a-chip devices for non-invasive and real-time tracking of biomolecules (*e.g.*, cytokines and cardiac-specific proteins). These technologies help clinicians identify changes as they happen, empowering preventive action to avert negative events. These innovations will help transition immunotherapy management from a reactive approach to proactive management.

With the ability to analyze massive heaps of data, AI also has the potential to revolutionize oncology by revealing trends and predicting outcomes. In immunotherapy, AI-based models combine patient data to stratify patients at risk for irAEs, such as cardiotoxicity, to direct treatment options. The prospects for AI in diagnostic imaging are especially exciting, as algorithms trained on imaging data can be utilized to detect subtle cardiac abnormalities through echocardiography or MRI-advancing the precision of their early detection and monitoring. Furthermore, AI-driven real-time analytics deriving from wearable devices,  such as smartwatches, enhance continuous monitoring of vital signs. This enables timely interventions and tailored suggestions for treatment modifications,  ultimately improving patient safety and outcomes.

As a subset of AI, ML is an integral part of tailoring immunotherapy to the individual by evaluating large data on how genetic components, treatment outcomes are interrelated to come up with a personalized regimen. Based on the analysis of biomarkers, imaging, and history, supervised learning predicts risks such as cardiotoxicity. In addition, ML has been successful in identifying novel irAE types and subtypes, which in turn can inform more focused diagnostics and interventions. It can maximize treatment protocols by simulating possible scenarios and leveraging patient-specific adaptation. Furthermore, ML facilitates real-time dynamic risk stratification by continuously updating a patient’s risk profile, which can guide clinicians in real-time adjustments of strategies further associated with risk reduction and improved outcomes.

Biomarkers in conjunction with AI and ML create a futuristic view of. For example, wearable biosensors can be used to give a reading of biomarker changes, while AI-powered platforms can analyze the data in real-time, and time-evolving risk assessment can be performed with ML algorithms. Startups are also accelerating drug development in this way by identifying safety issues upfront, simulating clinical trials, and optimizing therapeutic combinations to make treatment safer and more effective.

Especially with challenges posed by irAEs such as cardiotoxicity, it is likely that the future of cancer immunotherapy will be marked by transformative multi-faceted integration in which biomarkers, AI, and ML are used to broaden the sphere of immunotherapeutic benefits while minimizing toxicities. These technologies allow for a proactive and personalized patient care model, with capabilities for early detection, risk prediction, and dynamic treatment optimization. We believe that by incorporating these innovations into routine clinical practice, the field of oncology will help to secure the future of immunotherapy as a safe and efficacious pillar of cancer care and enter a new era of precision medicine.

## Figures and Tables

**Fig. (3):**
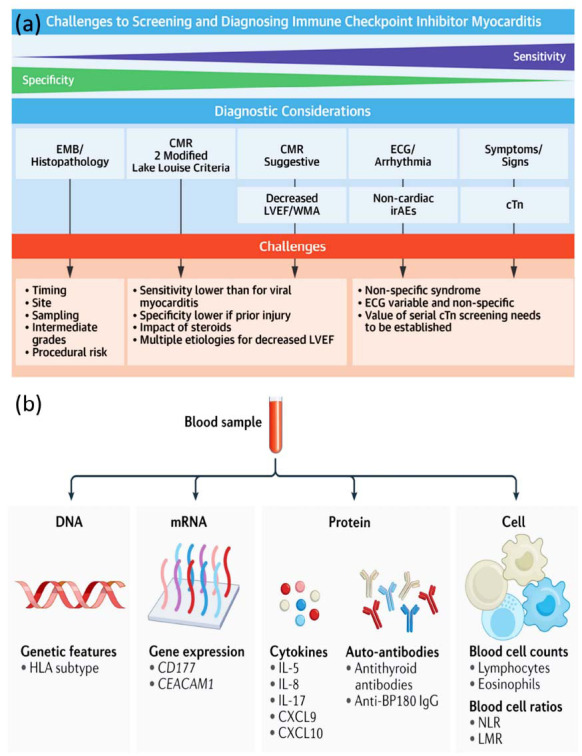
(**a**) Current challenges to diagnose and screen immune checkpoint inhibitor-induced myocarditis [76]; (**b**) Potential biomarkers identified from blood samples at different molecular levels, including DNA, mRNA, protein and cellular for early detection of cardiac irAEs [81]. **Abbreviations:** EMB: Endomyocardial Biopsy; CMR: Cardiac Magnetic Resonance; LVEF: Left Ventricular Ejection Fraction; WMA: Wall Motion Abnormalities; ECG: Electrocardiogram; cTn: Cardiac Troponin; irAEs: Immune-Related Adverse Events; HLA: Human Leukocyte Antigen; CD177: Cluster of Differentiation 177; CEACAM1: Carcinoembryonic Antigen-Related Cell Adhesion Molecule 1; IL-5, IL-8, IL-17: Interleukins 5, 8, and 17; CXCL9, CXCL10: Chemokine (C-X-C motif) Ligand 9 and 10; Anti-BP180 IgG: Autoantibody targeting BP180 (Bullous Pemphigoid Antigen 180) Immunoglobulin G; NLR: Neutrophil-to-Lymphocyte Ratio; LMR: Lymphocyte-to-Monocyte Ratio.

**Table 1:** Comparison of ICIs, chemotherapy, radiation therapies.

**Feature**	**Immune Checkpoint Inhibitors (ICIs)**	**Chemotherapy**	**Radiation Therapies**
Mechanism of action	ICIs block proteins like PD-1, PD-L1, and CTLA-4, reactivating T-cells to recognize and destroy cancer cells. Effective in cancers like melanoma, lung cancer, and renal cancer [5].	Chemotherapy uses cytotoxic drugs to kill rapidly dividing cells, both cancerous and healthy. Widely used for solid tumours and hematologic malignancies [6].	Radiation damages the DNA of cancer cells, inhibiting replication or causing cell death. Primarily for localized cancers, including breast, prostate, and brain cancers [7].
Target site	Targets immune checkpoints to enhance the immune system's ability to attack cancer indirectly. Restores immune surveillance to recognize and eradicate cancer cells [8].	Non-specific, affecting all rapidly dividing cells. Reduces tumour burden directly by killing cancer cells [9].	Targets cancer in localized areas but can affect nearby healthy tissues. Reduces or eliminates localized cancer and prevents spread [10].
Systemic effects	Provides systemic immune activation, enabling the treatment of metastatic cancers [11].	Systemic toxicity leads to side effects like myelosuppression, nausea, and fatigue [12].	Mostly localized effects, though systemic responses occur in cases like total-body irradiation [13].
Side effects	Immune-related adverse events [14].	Commonly causes nausea, vomiting, hair loss, and increased infection risk [15].	Side effects depend on the treated area, including burns, fibrosis, or fatigue [16].
Cost	High due to advanced technologies and testing.	Generally less expensive than ICIs but costly due to multiple cycles.	Costs depend on technology.
Cardiotoxicity	Myocarditis, vasculitis, pericarditis (20–45% mortality)	Heart failure, arrhythmias (dose-related).	Rare, mostly due to exposure near the heart.

## LIST OF ABBREVIATIONS

AI: Artificial Intelligence
ICIs: Immune Checkpoint Inhibitors
irAEs: Immune-related Adverse Events
MHC: Major Histocompatibility Complex
ML: Machine Learning
MRI: Magnetic Resonance Imaging
PFS: Progression-free Survival
